# Does the Ovarian Stimulation Phase Length Predict *In vitro* Fertilization Outcomes?

**Published:** 2011-12-22

**Authors:** Brie Alport, Allison Case, Hyun Lim, Angela Baerwald

**Affiliations:** 1 Department of Obstetrics, Gynecology and Reproductive Sciences, Division of Reproductive Endocrinology and Infertility, Royal University Hospital, College of Medicine, University of Saskatchewan, 103 Hospital Drive, Saskatoon, Saskatchewan, Canada, S7N 0W8; 2 Department of Community Health and Epidemiology, Royal University Hospital, College of Medicine, University of Saskatchewan, 103 Hospital Drive, Saskatoon, Saskatchewan, Canada, S7N 0W8

**Keywords:** Ovarian Stimulation Phase, IVF, Oocyte, Embryo, Pregnancy

## Abstract

**Background:**

Bi-directional communication between the follicle and oocyte is necessary to regulate follicle and oocyte development. Currently, it is not practical to monitor the serial growth of individual follicles during assisted reproduction. The ovarian stimulation phase length (SPL) is an indirect measure of mean follicular growth rate. The objective of this study was to test the hypothesis that a short or long SPL would be associated with suboptimal outcomes in women undergoing *in vitro* fertilization (IVF).

**Materials and Methods:**

A retrospective cohort study was conducted in 140 women who underwent IVF. Follicle development was monitored every 2-3 days during ovarian stimulation using transvaginal ultrasonography. Once > 3 follicles reached ≥ 17 mm, human chorionic gonadotropin (hCG) was administered. Oocyte retrieval was performed approximately 35 hours after hCG. Oocytes underwent IVF on the day of collection and were evaluated daily thereafter. Embryos were transferred on days 3 or 5, depending on the number and quality of embryos available. Associations between SPL, age, follicle, oocyte, embryo and pregnancy outcomes were evaluated (SPSS version 17.0; SPSS Inc., Chicago, IL, USA).

**Results:**

A SPL of 11 days was associated with an optimal number of follicles that developed to ≥ 6 mm, ≥ 10 mm and ≥ 15 mm; serum estradiol concentrations; and number of oocytes collected (p<0.05). Gradual reductions in the number of developing follicles, serum estradiol concentrations and number of oocytes collected occurred with SPL less than or greater than 11 days (p<0.05). The SPL did not influence endometrial, embryo or pregnancy outcomes (p>0.05). Associations between SPL and outcomes were not influenced by age (p>0.05).

**Conclusion:**

The ovarian SPL can be used to predict the number of follicles that develop, oocytes collected and serum estradiol concentrations, but not embryo or pregnancy outcomes.

## Introduction

Bi-directional communication between the follicle and oocyte is essential for regulating both follicle and oocyte development (1, 2). Knowledge about follicle-oocyte interactions has important implications for identifying biologic markers that predict the oocyte’s ability to be fertilized and develop into a healthy embryo and baby in women undergoing assisted reproduction. The identification of non-invasive markers of oocyte competence would reduce the incidence of multiple embryo transfer which would, in turn, reduce the incidence of multiple pregnancies and associated maternal-fetal risks.

At present, follicle diameter and serum estradiol levels are used as the primary markers for determining the maturity of the follicle and oocyte prior to oocyte retrieval and *in vitro* fertilization (IVF) (3, 4). Perifollicular vascularity has also been shown to be a good predictor of follicle maturity and oocyte/ embryo/pregnancy outcomes (5). It has been further suggested that the growth profiles of the developing follicles may be important in predicting pregnancy potential (6-11). The mean growth rate of follicles during the natural menstrual cycle has been reported to be quite variable, between 1-4 mm/day (6, 9, 10, 12, 13). The mean follicular growth rate during ovarian stimulation cycles has been reported to be 1.7 mm/day, which was greater than that during the spontaneous menstrual cycle (1.4 mm/day) (6). The potential effects of greater follicular growth rates on the development of the oocyte, embryo and resulting pregnancy in women undergoing ovarian stimulation are currently unknown.

Zegers-Hochschild et al. reported that stabilization of follicular growth 24 hours prior to ovulation and a short interval (i.e., 24 hours or less) between the luteinizing hormone (LH) surge and ovulation was associated with conception during the natural menstrual cycle (11). It has been suggested that the rate of early follicular growth during ovarian stimulation is most predictive of pregnancy potential, with slow early follicular growth being predictive of negative pregnancy outcomes (9). Others have reported that a short follicular phase during an intrauterine insemination (IUI) cycle, occurring in association with early ovulation (i.e., before day 11), is associated with poor pregnancy potential compared to a long follicular phase (14). These early studies have been an important step in determining how follicle development influences oogenesis and embryogenesis in couples undergoing IVF or intracytoplasmic sperm injection (ICSI). However, results of studies are contradictory. Results obtained thus far are limited because researchers employed lowresolution transabdominal ultrasonography to monitor follicular development (as opposed to currently used transvaginal ultrasonography), older hormonal stimulation therapies were administered with no comparisons between regimens and small samples of women were evaluated with no consideration of age.

A decrease in the number of follicles occupying the ovarian reserve occurs with age (15). An agerelated increased incidence of oocyte aneuploidy and a corresponding increased risk of spontaneous abortion have been well documented in women (16). As women enter the transition to menopause, the length of the menstrual cycle decreases (17). The shortened cycles in older ovulatory women have been attributed to early selection of the dominant follicle and thus a shortened follicular phase (18). Earlier selection of the dominant ovulatory follicle in aging women has been attributed to either a faster growth rate of the dominant follicle (19) or earlier emergence of the follicular cohort in the late luteal phase of the preceding cycle (20). The decreased length of the follicular phase in older reproductive-age women corresponds to lower inhibin B and higher follicle stimulating hormone (FSH) levels in the early follicular phase, while estrogen levels appear to remain the same or are slightly decreased (21-29). Success rates of IVF/ ICSI have been shown to correlate strongly with mean menstrual cycle length, independent of age (30). However, it is not currently known whether age-related changes in follicular growth rates during ovarian stimulation influence IVF outcomes.

It is not practical, at present, to monitor the serial growth of individually identified follicles in women undergoing assisted reproduction. The ovarian stimulation phase length (SPL) is a user-friendly way for clinicians to monitor the overall growth of all follicles and serves as an indirect measure of mean follicular growth rate. Determination of an optimal SPL that results in competent oocytes and successful IVF outcomes would allow clinicians to tailor patient stimulation protocols to maximize the chance of a successful treatment. The objective of this study was to determine whether SPL influences follicle, oocyte, embryo and pregnancy outcomes in women of different ages undergoing IVF. We hypothesized that short or long stimulation phases would be associated with suboptimal follicle, oocyte, embryo and pregnancy outcomes. We further hypothesized that advanced age would be associated with a shorter stimulation phase and suboptimal follicle, oocyte, embryo and pregnancy outcomes.

## Materials and Methods

It is not practical or ethical to conduct a prospective study to evaluate the effects of different ovarian stimulation phase lengths on human IVF outcomes. Therefore, a retrospective cohort study was conducted for patients that underwent IVF at the ARTUS Fertility Center in the Department of Obstetrics, Gynecology and Reproductive Science at the University of Saskatchewan. Ethical approval was obtained from the University of Saskatchewan’s Biomedical Research Ethics Board.

Women with a history of poor response to ovarian stimulation, polycystic ovarian syndrome, those undergoing superovulation (SO) or ICSI treatment cycles, or who had converted from SO to IVF were not included. In addition, frozen embryo transfer cycles, oocyte donor cycles and couples with male factor infertility were excluded from the study. A total of 148 charts were reviewed. A total of 14 women were further excluded from the study for the following reasons: inadequate follicular response (n=12), premature ovulation (n=1) and unavailable participant semen sample (n=1). The resulting sample consisted of 134 women.

### Ultrasonographic assessment of ovarian follicular development

All patients were cared for by the same clinician. Follicular development was monitored every 2-3 days during ovarian stimulation using high-resolution transvaginal ultrasonography (multi-frequency 5-9 MHz curvilinear transducer, SONIX OP Ultrasound System, Ultrasonix Medical Corporation, Burnaby, BC Canada). All follicles ≥2 mm were measured and recorded at each ultrasound examination. The diameters of follicles <10 mm were measured in a single plane. For all follicles >10 mm, mean maximal follicle diameter was calculated as the mean of the follicle length and width in the widest plane of section.

### Ovarian stimulation

All patients were suppressed with oral contraception (OC; Marvelon, Organon, Canada), prior to ovarian stimulation to synchronize their cycles (n=134). Patients received either a gonadotropinreleasing hormone (GnRH) agonist [Suprefact, Sanofi-Aventis (n=56)] for 5 days before discontinuing OCs and continuing until the day of hCG (i.e., long GnRH agonist protocol) or a GnRH antagonist [(Cetrotide, EMD Serono, Inc. (n=62); Orgalutran, Organon, Canada (n=16)] beginning on day 6 of the follicular phase and continuing until the day of hCG (i.e., fixed protocol). Recombinant FSH [Gonal F, Serono, Canada, Inc. (n=100); Puregon, Organon, Canada (n=18); Bravelle, Ferring Pharmaceuticals, Inc. (n=11); or Menopur, Ferring Pharmaceuticals, Inc. (n=5)] was administered daily, beginning on day 2 or 3 after menses. When > 3 follicles reached >17 mm, 10,000 IU hCG (Profasi, Serono Canada Inc.; Chorionic Gonadotropin, Pharmaceutical Partners of Canada) was administered to induce final maturation of the follicles. Blood was drawn at each monitoring visit during ovarian stimulation to measure serum estradiol concentrations. The SPL was defined as the time period from the start of follicle stimulating hormone (FSH) administration to the day of hCG administration.

### In vitro fertilization

Oocyte retrievals were performed approximately 35 hours following hCG administration. Oocytes were inseminated (standardized sperm concentration of 80-120 × 10^6^ sperm/mL) 4 hours following retrieval. Fertilization was assessed the next day and daily embryo monitoring was conducted thereafter. Fertilization rate was defined as the number of normally fertilized oocytes (release of the second polar body and two pronuclei) out of the total number of oocytes collected. Cleavage rate was characterized by the number of zygotes that underwent division on day 2 out of the total number of oocytes fertilized (day 0 = day of oocyte retrieval). Blastocyst rate was defined as the number of blastocysts that developed out of the total number of oocytes fertilized. An appropriate number of embryos [1 embryo (n=13), 2 embryos (n=100) or 3 embryos (n=27)] were transferred 3 or 5 days later; based on patient age, diagnosis, previous treatment success, and number and quality of embryos available for transfer. A serum β-hCG test was performed 2 weeks post-retrieval. Chemical pregnancy was defined as a positive serum pregnancy test 2 weeks following oocyte retrieval. Clinical pregnancy was documented ultrasonographically as a positive fetal heartbeat at 8-12 weeks of gestation. Live birth data were not available due to the inability to consistently obtain birth outcome results from patients.

### Statistical analyses

SPL was considered the independent variable. Pregnancy rate (chemical and clinical) was the primary dependent outcome variable. Secondary outcome variables included: number of follicles > 6, 10 and 15 mm, serum estradiol concentration on the day of hCG, number of oocytes collected, fertilization rate, cleavage rate, blastocyst rate and endometrial thickness. Statistical significance was set at p<0.05.

SPL data were stratified in the following manner: 1) <10 days, 2) 10-12 days, and 3) >12 days. Patient demographic characteristics were compared among the three SPL groups using one-way analyses of variance tests and Scheffe post-hoc tests for continuous variables and chi-square tests for categorical variables (SPSS version 17.0; SPSS Inc., Chicago, IL, USA). Associations between SPL and primary/ secondary outcomes were evaluated using multivariate linear and logistic regression (SPSS Version 17.0; SAS Version 9.2). Age, FSH start day, FSH start dose, FSH regimen and GnRH agonist/antagonist use were included in the multivariate regression models to evaluate these variables as potential confounders or covariates.

## Results

No differences in body mass index (BMI), gravidity, parity or age were detected between women with short, medium or long SPL (p>0.05, Table 1).

**Table 1:** Comparison of patient demographics in women with short (<10 days), moderate (10-12 days) and long (>12 days) stimulation phase lengths


		Overall	<10 days (n=18)	10 to 12 days (n=101)	>12 days (n=15)	P value

**BMI (mean ± SE)**		25.3 ± 0.5	25.2 ± 1.4	25.6 ± 0.6	23.9 ± 1.3	0.6
**Gravidity (mean ± SE)**		1.5 ± 0.1	1.9 ± 0.5	1.3 ± 0.2	1.8 ± 0.5	0.4
**Parity (mean ± SE)**		0.7 ± 0.1	0.6 ± 0.2	0.6 ± 0.1	1.1 ± 0.3	0.2
**Age (mean ± SE)**		33.8 ± 0.4	33.8 ± 1.1	33.8 ± 0.4	33.6 ± 1.1	1.0
**Smoking (n)**	**Yes**	6	2^a^	4^b^	0^a^	0.04
	**No**	128	16^a^	97^b^	15^a^	


^a, b^ Within rows, values with common superscripts are not different (p>0.05).

**Fig 1:**
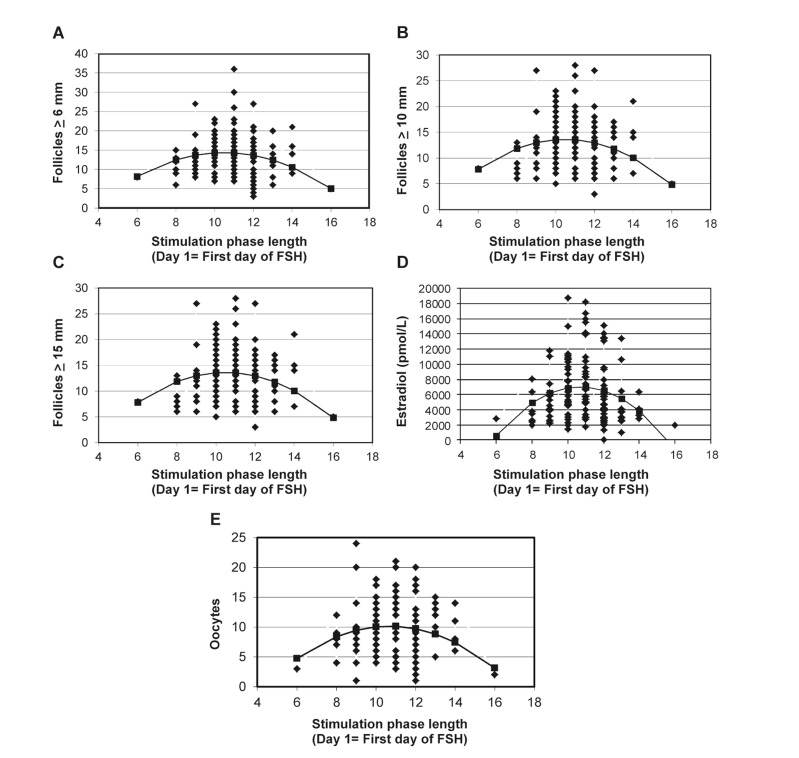
Associations between the stimulation phase length (SPL) and number of follicles that developed to ≥ 6, 10 and 15 mm (A-C, respectively), serum estradiol concentrations (D) and the number of oocytes retrieved (E). The solid line on each graph represents the best fit regression line.

**Fig 2:**
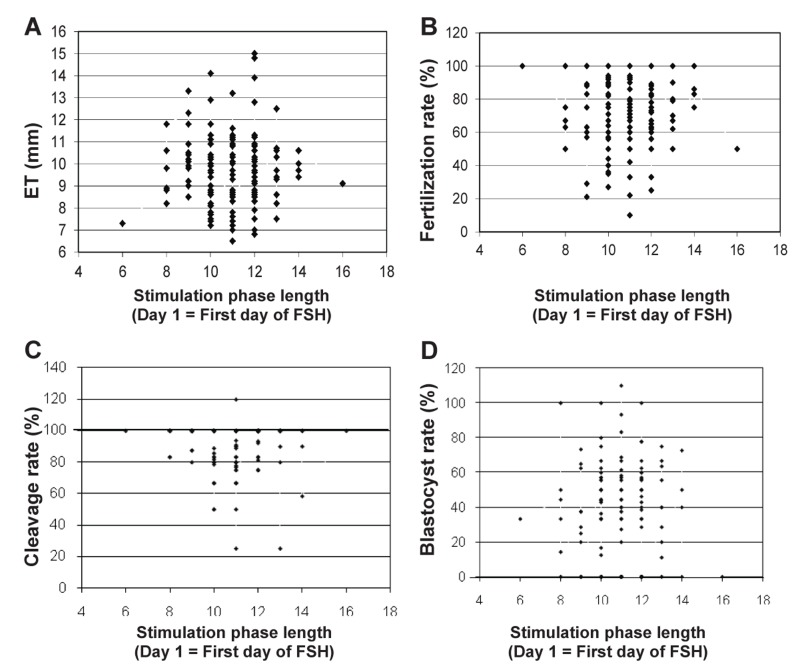
Associations between the stimulation phase length (SPL) and endometrial thickness (ET), fertilization rate (%), cleavage rate (%) and blastocyst rate (%). No regression lines are shown because no associations were detected.

**Table 2:** Confounding variables for the associations between stimulation phase length (SPL) and IVF outcomes


	P-value	SE (β)	Estimate (β)	Model

**No. follicles >6 mm**	SPL	7.19	2.56	0.006
	SPL^2^	-0.34	0.12	0.005
	Confounder=FSH start dose	-0.02	0.005	<0.0001
**No. follicles >10 mm**	SPL	6.77	2.50	0.008
	SPL^2^	-0.32	0.11	0.005
	Confounder=FSH start dose	-0.02	0.004	0.0002
**No. follicles >15 mm **	SPL	4.92	1.74	0.005
	SPL^2^	-0.23	0.08	0.004
	Confounder=FSH start dose	-0.008	0.003	0.01
**No. oocytes retrieved**	SPL	5.67	2.28	0.014
	SPL^2^	-0.26	0.10	0.013
	Confounder=FSH start dose	-0.009	0.004	0.021
**Peak estradiol (pmol/L)**	SPL	5714	1778.9	0.002
	SPL^2^	-263	81.2	0.002
	Confounder=GnRH agonist vs. antagonist	-3561	607.8	<0.0001


The following variables were not found to be associated with SPL (SPL, SPL^2^; p>0.05): endometrial thickness, fertilization rate, cleavage rate, blastocyst rate, chemical pregnancy rate and clinical pregnancy rate. Results were obtained using multivariate polynomial linear regression analyses. SPL = Stimulation phase length (linear relationship) SPL^2^ = Stimulation phase length (parabolic relationship)

There was a greater incidence of non-smokers versus smokers within all 3 groups (p<0.05). The incidence of smokers was greater in women with a moderate versus a short or long SPL (p=0.04).

Negative parabolic associations were detected between SPL and the number of follicles that developed to ≥ 6, 10 and 15 mm, number of oocytes collected, and serum estradiol concentrations on the day of hCG (Table 2, Fig 1). That is, the number of follicles that developed, serum estradiol concentrations and number of oocytes retrieved peaked at a SPL of 11 days and declined progressively with shorter or longer phase lengths. No associations were detected between SPL and endometrial thickness, fertilization rate, cleavage rate or blastocyst rate (p>0.05, Fig 2). Similarly, SPL was not associated with the chemical (p=0.99) or clinical pregnancy rate (p=0.97).

After adjusting for all potentially confounding variables in our multivariate regression models, we found that the number of follicles that developed to ≥ 6, 10 and 15 mm and number of oocytes collected was negatively confounded by the FSH stimulation start dose (p<0.05, Table 2). In other words, the regression line for follicles that developed (in all 3 diameter categories) was lower throughout the ovarian stimulation cycle when a higher FSH start dose was used. Furthermore, serum estradiol concentration on the day of hCG was confounded by the use of GnRH agonist versus antagonist regimes (p<0.0001, Table 2). That is, the regression line for estradiol concentration was lower overall in women who used antagonist versus agonist therapy.

**Fig 3:**
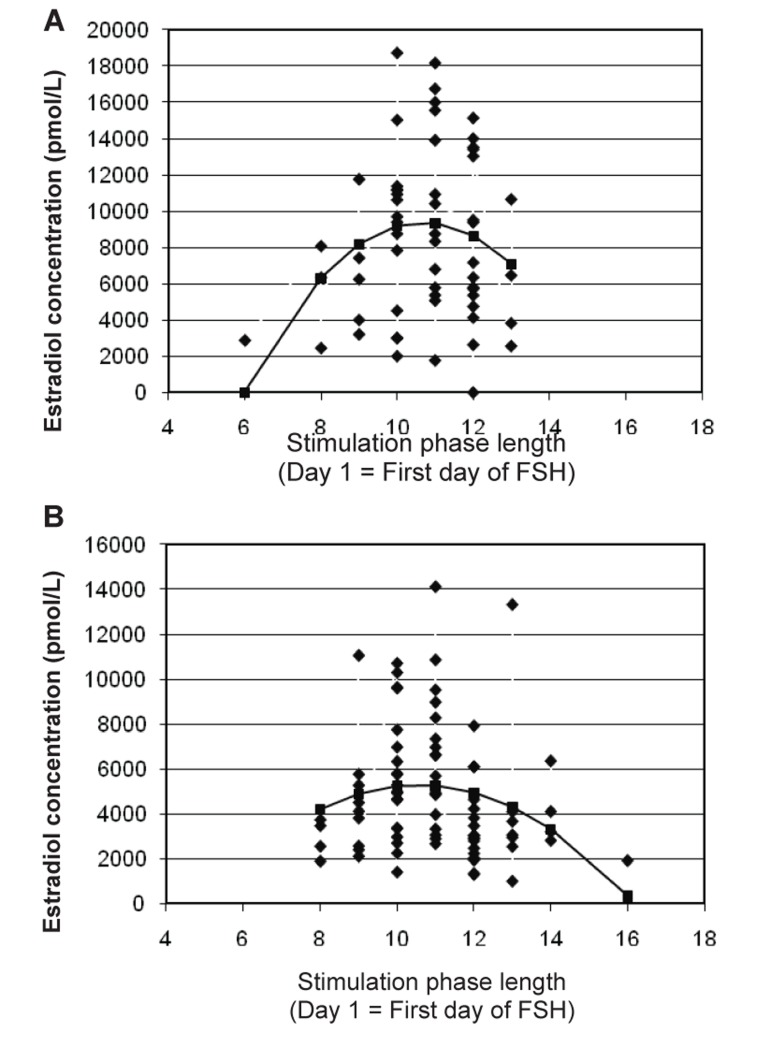
Associations between SPL and serum estradiol concentrations (D), stratified for GnRH agonist (A) versus antagonist (B) cycles. The solid line on each graph represents the best fit regression line.

## Discussion

It is not practical to monitor the growth of individual follicles that develop during ovarian stimulation, using current clinical assisted reproductive practices. We have conducted the present study to determine whether the duration of ovarian stimulation could be used as a reliable marker of mean ovarian follicular growth to predict clinical outcomes in couples undergoing IVF. The results of the present study have indicated that the length of the ovarian stimulation phase can be used to predict the number of estrogen-producing follicles that develop and oocytes retrieved prior to IVF. However, endometrial, fertilization, embryo and pregnancy outcomes were not influenced by the SPL. Thus, our hypotheses were only partially supported.

Increasing the FSH dose in patients whose follicles are growing slowly or decreasing the FSH dose in patients whose follicles are developing very quickly to achieve a SPL of approximately 11 days should therefore optimize follicular and oocyte outcomes, but not increase the probability of fertilization, high quality embryos or pregnancy. Collectively, we interpret these data to mean the SPL is not a reliable indicator of IVF success.

The effects of age on menstrual cycle length, conception and delivery rates are well-documented; however, potential age-related changes in the length of the SPL are not fully understood. We did not find that age had an effect on the SPL nor did it appear to confound the association between SPL and study outcomes. Thus, our hypothesis that women of advanced age would exhibit shorter SPLs and poorer clinical outcomes was not supported. Data on follicular phase length during previous natural cycles were not available in the present study. It is plausible that an age-related shortening of the follicular phase during the natural menstrual cycle does not necessarily correspond to a decreased SPL during ovarian stimulation. Continued research should be conducted to test this hypothesis.

In addition to age, we studied the potentially confounding effects of the ovarian stimulation hormonal treatment regimen on the association between SPL and IVF outcomes. A high FSH start dose was associated with an overall reduction in the number of follicles that developed. However, the pattern of change in follicle number in relation to SPL did not differ with different FSH start doses. The negative association between the FSH start dose and the number of follicles that developed was attributed to a higher dose of FSH that may have been administered to women expected to have a poor response to stimulatory therapy (even though women with a known history of a poor response were excluded from our analyses).

In addition, we found that the use of GnRH antagonist was strongly associated with lower overall estradiol concentrations throughout the stimulation phase compared to the use of agonists. The pattern of change in estradiol concentrations in relation to SPL, however, was not different in agonist versus antagonist cycles. Our findings were consistent with previous research that demonstrated lower serum estradiol concentrations in women using GnRH antagonists versus agonists during ovarian stimulation (31-33).

The ability to obtain more mature follicles and oocytes by optimizing the SPL, but not more embryos and pregnancies, supports the notion that follicle quality is more important than follicle quantity in predicting the probability of pregnancy following IVF. These results are consistent with those from a previous study in which the number of dominant follicles during ovarian stimulation was not associated with pregnancy success (32). It is plausible that the serial growth characteristics (i.e., growth rate, changes in vascularity) of individual follicles influence the physiologic status of corresponding oocytes and embryos. Thus, future research should focus on developing practical methods for tracking the growth of individually-identified follicles that develop over the course of ovarian stimulation. The development of such a tool could assist in identifying follicle-specific physiologic, genomic, proteomic or metabolic markers of human oocyte and embryo competence, which may ultimately increase the likelihood of achieving a healthy baby in couples undergoing assisted reproduction.

## Conclusion

A short or long ovarian stimulation phase length is associated with a suboptimal number of follicles that develop, serum estradiol concentrations and number of oocytes retrieved in couples undergoing assisted reproduction. However, the length of the stimulation phase does not predict embryo development or pregnancy outcomes.
